# Synthesis and Characterization of CuIn_1−*x*_Ga*_x_*Se_2_ Semiconductor Nanocrystals

**DOI:** 10.3390/nano10102066

**Published:** 2020-10-19

**Authors:** Yu-Tai Shih, Yu-Ching Tsai, Der-Yu Lin

**Affiliations:** 1 Department of Physics, National Changhua University of Education, Changhua 50007, Taiwan; m0423010@gm.ncue.edu.tw; 2 Department of Electronic Engineering, National Changhua University of Education, Changhua 50074, Taiwan; dylin@cc.ncue.edu.tw

**Keywords:** CuIn_1−*x*_Ga*_x_*Se_2_, nanocrystals, chalcopyrite, Vegard’s law, band gap energy, Raman spectra

## Abstract

In this paper, the synthesis and characterization of CuIn_1−*x*_Ga*_x_*Se_2_ (0 ≤ *x* ≤ 1) nanocrystals are reported with the influences of *x* value on the structural, morphological, and optical properties of the nanocrystals. The X-ray diffraction (XRD) results showed that the nanocrystals were of chalcopyrite structure with particle size in the range of 11.5–17.4 nm. Their lattice constants decreased with increasing Ga content. Thus, the *x* value of the CuIn_1−*x*_Ga*_x_*Se_2_ nanocrystals was estimated by Vegard’s law. Transmission electron microscopy (TEM) analysis revealed that the average particle size of the nanocrystals agreed with the results of XRD. Well-defined lattice fringes were shown in the TEM images. An analysis of the absorption spectra indicated that the band gap energy of these CuIn_1−*x*_Ga*_x_*Se_2_ nanocrystals was tuned from 1.11 to 1.72 eV by varying the *x* value from 0 to 1. The Raman spectra indicated that the A_1_ optical vibrational mode of the nanocrystals gradually shifted to higher wavenumber with increasing *x* value. A simple theoretical equation for the A_1_ mode frequency was proposed. The plot of this equation showed the same trend as the experimental data.

## 1. Introduction

In recent years, due to issues of global warming, the exhaustion of traditional and nonrenewable energy sources, and the goal of gradually reducing nuclear power generation, the demand for clean, safe, and renewable energy around the world has increased significantly. The research and development of photovoltaic devices have attracted great interest and attention.

The earliest photovoltaic devices were made of crystalline silicon, which is currently the main material for the mass production of solar cell modules. However, due to high production costs, many researchers have turned to other photovoltaic materials in order to replace crystalline silicon. Among them, CuIn_1−*x*_Ga*_x_*Se_2_ has received extensive attention.

CuIn_1−*x*_Ga*_x_*Se_2_ is a quaternary semiconductor material belonging to the family of I–III–VI_2_ compounds [1]; it has a tetragonal chalcopyrite structure. CuIn_1−*x*_Ga*_x_*Se_2_ has good optical properties, such as intrinsic high optical absorption coefficient (α ≈ 10^5^/cm), wide absorption range, good radiation stability [2,3,4,5], and a direct bandgap which is adjustable by changing the composition ratio of In to Ga. For this reason, it is regarded as an excellent absorber layer material for thin-film solar cell applications [6,7]. A recent report indicated that the conversion efficiency of CuIn_1−*x*_Ga*_x_*Se_2_ solar cells has reached 22.6% [8]. Additionally, CuIn_1−*x*_Ga*_x_*Se_2_ could be a promising material for other optoelectronic applications because its composition tunability opens another parameter with which to achieve specific properties and performance [9].

Generally, the absorber layers of solar cells are manufactured by vacuum-based processes, which have the disadvantages of complicated procedures, problems in scale-up, and high production costs. A promising approach for improvement is using semiconducting nanocrystals in the manufacturing processes of solar energy harvesting devices [10,11,12,13,14]. Nanocrystal-based printing/coating technology for the absorber layers of photovoltaic devices can not only reduce production costs, but also achieve high efficiency [15,16]. Research results have shown that CuIn_1−*x*_Ga*_x_*Se_2_ nanocrystals are a good absorber layer material for thin-film solar cell applications [4,5,17].

There are various methods for manufacturing CuIn_1−*x*_Ga*_x_*Se_2_ nanocrystals, such as hot injection synthesis [5,18], the solvothermal route [4], thermal decomposition [19,20,21], the modified polyol route [22], and the mechanochemical process [23]. Cui and co-workers synthesized CuIn_1−*x*_Ga_*x*_Se_2_ nanocrystals by heating metal chlorides in a selenium solution containing oleylamine and glycerol [24]. Recently, two-step procedures, which include a low temperature (110 °C) nucleation stage lasting 24 h followed by a growth step at higher temperature (240 °C) lasting 1 h, to synthesize CuIn_1−*x*_Ga*_x_*Se_2_ nanocrystals with sphalerite or wurtzite phase were reported [25]. In this paper, we report the synthesis of CuIn_1−*x*_Ga_x_Se_2_ nanocrystals over the entire nominal composition range (0 ≤ *x* ≤ 1) by a solution-based method [26]. Due to the low reaction temperature and short reaction time, this method is one of the simplest synthesis methods for manufacturing CuIn_1−*x*_Ga*_x_*Se_2_ nanocrystals, and is practical for mass production. In addition, this method has the potential to be environmentally friendly and reduce costs, as there is no need for toxic gases, high vacuum or high temperature facilities.

In this study, the band gap energy of the CuIn_1−*x*_Ga_x_Se_2_ nanocrystals was tuned by varying the *x* value. The influences of *x* value on the structural, morphological, and optical properties of the nanocrystals were studied. The nanocrystals could form colloidal suspensions that are paving the way for the fabrication of photovoltaic devices.

## 2. Materials and Methods

To synthesize CuIn_1−*x*_Ga*_x_*Se_2_ nanocrystals over the entire nominal composition range, a useful solution-based method was developed [26]. The nominal *x* values were 0, 0.125, 0.25, 0.375, 0.5, 0.625, 0.75, 0.875, and 1. In a typical preparation procedure, 1 mmol (0.2618 g) of copper(II) acetylacetonate, (1–*x*) mmol (0.4121(1–*x*) g) of indium(III) acetylacetonate, *x* mmol gallium(III) acetylacetonate (0.3671*x* g), and 3.5 mmol (1.3532 g) of trioctylphosphine oxide were added to a four-neck, round bottom flask containing 10 mL of oleylamine. The mixture was magnetically stirred at room temperature for 30 min and then heated to 150 °C. Afterwards, 2 mmol (0.1579 g) of elemental selenium powder was added to the solution with continuous stirring. The reaction solution was then heated to 230 °C and maintained at this temperature for 30 min with stirring, and then cooled to room temperature. During the synthesis procedure, the reaction was performed under a nitrogen atmosphere. Next, a mixture of 25 mL of hexane and 25 mL of ethanol was added to the solution. The precipitates of the crude solution were collected by centrifugation at 7000 rpm for 15 min. Finally, the precipitated nanocrystals were dissolved into a suitable amount of chloroform and then centrifuged at 7000 rpm for 15 min. This process was repeated two times to obtain high-purity nanocrystals.

The structural properties of the CuIn_1−*x*_Ga*_x_*Se_2_ nanocrystals were analyzed by X-ray diffraction (XRD) using a Shimadzu XRD-6000 (Kyoto, Japan) diffractometer equipped with Fe Kα radiation. Transmission electron microscope (TEM) images of the nanocrystals were taken using a JEOL JEM-2010 (Tokyo, Japan) transmission electron microscope. For absorption measurements, a 1/4 m monochromator (MKS, Irvine, CA, USA) equipped with a 130 W halogen lamp was used to provide monochromatic light in a photon energy range from 0.9 to 1.8 eV. The continuous light coming from the monochromator was modulated into an alternate light at a frequency of 200 Hz through a mechanical chopper. A silicon photo detector (Thorlabs, Newton, NJ, USA) and an InGaAs photo detector (Thorlabs, Newton, NJ, USA) with an amplifier were placed on the back of the sample to detect the intensity of the transmitting light. The output signal of the detector was recorded by a dual phase lock-in amplifier (Ametek, Berwyn, PA, USA) to suppress noise signals. Raman analyses were performed using a Dongwoo Ramboss Micro Raman (Gwangju-Si, Korea) system with a solid state laser source with an excitation wavelength of 532 nm.

## 3. Results and Discussion

### 3.1. Structural Properties

**Figure 1:**
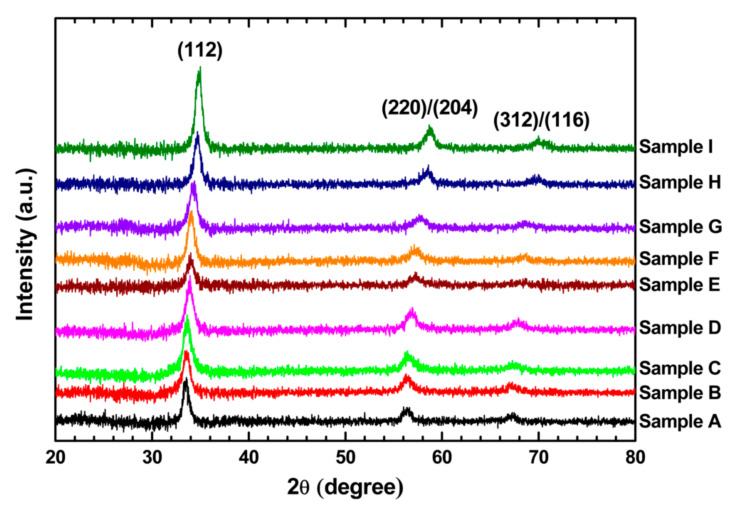
X-ray diffraction patterns of CuIn_1−*x*_Ga*_x_*Se_2_ nanocrystals.

Figure 1 shows the XRD patterns of the CuIn_1−*x*_Ga*_x_*Se_2_ nanocrystals prepared in this work. Compared with the standards of the International Centre for Diffraction Data (ICDD) for CuInSe_2_ (JCPDS 01-087-2265) and CuGaSe_2_ (JCPDS 01-075-0104), it can be confirmed that all nanocrystals exhibited tetragonal structures with space groups of I-42d, i.e., chalcopyrite structures. For each XRD spectrum, three diffraction peaks appeared at 2*θ* around 34°, 56°, and 67°, representing the (112), (220)/(204), and (312)/(116) lattice planes of a chalcopyrite structure, respectively. No additional peaks were found in these patterns, implying the high phase purity of the materials. With an increase in the Ga content, the diffraction peaks shifted towards higher 2*θ* values. This was due to the changes in the lattice parameters *a* and *c*. From these XRD spectra, the distance *d_hkl_* between neighboring (*hkl*) planes was calculated by Bragg’s diffraction Equation:

(1) $$2d_{hkl}\sin\theta_{hkl}=n\lambda ,$$

where *n* is a positive integer, *λ* (=1.936 Å) is the wavelength of the X-ray and *θ_hkl_* is Bragg’s angle corresponding to the (*hkl*) planes. The lattice parameters *a* and *c* were then calculated according to the formula:

(2) $$d_{hkl}=\frac{1}{\sqrt{\frac{h^{2}+k^{2}}{a^{2}}+\frac{l^{2}}{c^{2}}}}.$$

Table 1 shows the calculated values of *d*_112_, *a*, and *c* from the XRD spectra. These values decrease as the Ga content is increased. Since the ionic size of Ga (0.62 Å) is smaller than that of In (0.81 Å) [27], the replacement of In by Ga leads to a decrease of interatomic distances. Table 1 also indicates that the value of *c*/*a* is very close to 2 for every sample, which means that the distortions of the tetragonal lattices are negligible in the CuIn_1−*x*_Ga*_x_*Se_2_ nanocrystals [28].

For alloy crystals, Vegard’s law predicts that their lattice parameters are approximately a weighted mean of the lattice parameters of their two constituents. Many reports have shown that Vegard’s law still works for nanocrystals [4,14,29,30,31]. Hence, the prospective lattice constants of the CuIn_1−*x*_Ga*_x_*Se_2_ nanocrystals can be expressed in the following forms:

(3) $$\left\{\begin{array}{l} a_{{\mathrm{CuIn}}_{1-x}{\mathrm{Ga}}_{x}{\mathrm{Se}}_{2}}=(1-x)a_{{\mathrm{CuInSe}}_{2}}+xa_{{\mathrm{CuGaSe}}_{2}},\\ c_{{\mathrm{CuIn}}_{1-x}{\mathrm{Ga}}_{x}{\mathrm{Se}}_{2}}=(1-x)c_{{\mathrm{CuInSe}}_{2}}+xc_{{\mathrm{CuGaSe}}_{2}}. \end{array}\right.$$

Accordingly, a reasonable *x* value for the *i*th sample with lattice constants *a_i_* and *c_i_*, which are determined from the XRD results, can be estimated by minimizing

(4) $$f(x)={\left(\frac{a_{i}-a_{{\mathrm{CuIn}}_{1-x}{\mathrm{Ga}}_{x}{\mathrm{Se}}_{2}}}{a_{{\mathrm{CuInSe}}_{2}}-a_{{\mathrm{CuGaSe}}_{2}}}\right)}^{2}+{\left(\frac{c_{i}-c_{{\mathrm{CuIn}}_{1-x}{\mathrm{Ga}}_{x}{\mathrm{Se}}_{2}}}{c_{{\mathrm{CuInSe}}_{2}}-c_{{\mathrm{CuGaSe}}_{2}}}\right)}^{2}.$$

**Figure 2:**
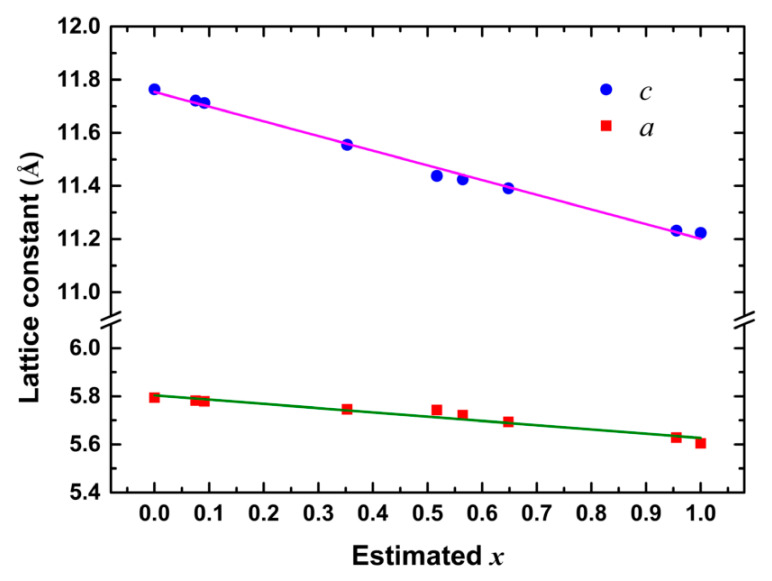
Lattice constants of CuIn_1−*x*_Ga*_x_*Se_2_ nanocrystals as functions of estimated *x*.

Table 1 shows the estimated *x* value for every sample. The lattice constants *a* and *c* of these CuIn_1−*x*_Ga*_x_*Se_2_ samples are plotted in Figure 2 as functions of the estimated *x* value. They are approximately linear as *x* increases from 0 to 1.

**Figure 3:**
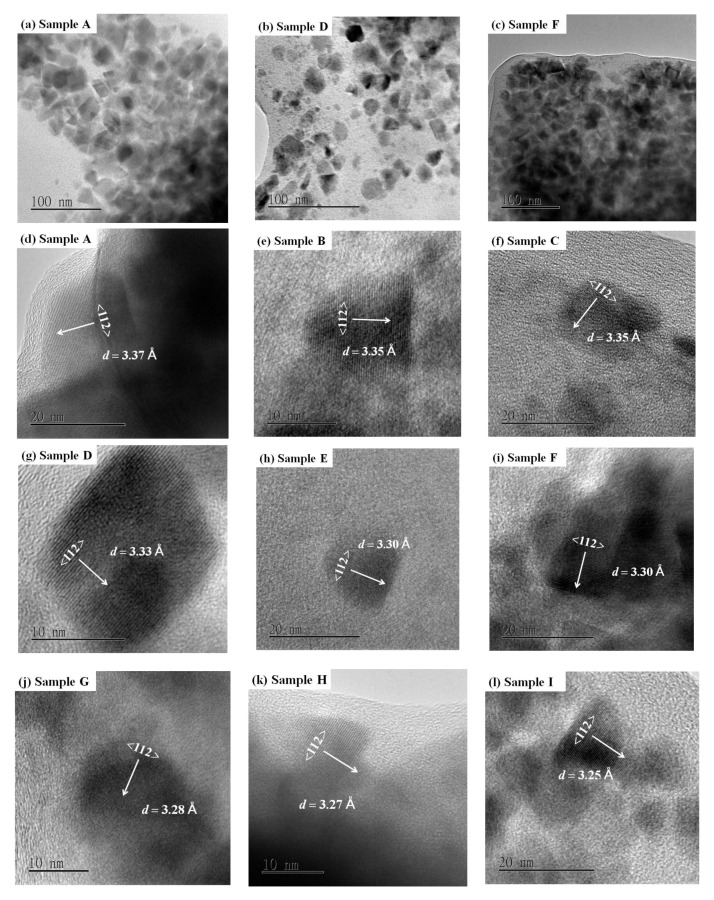
TEM images of CuIn_1−*x*_Ga*_x_*Se_2_ nanocrystals: (**a**) Sample A, (**b**) Sample D, (**c**) Sample F, (**d**) Sample A, (**e**) Sample B, (**f**) Sample C, (**g**) Sample D, (**h**) Sample E, (**i**) Sample F, (**j**) Sample G, (**k**) Sample H, (**l**) Sample I.

The broadening in the diffraction peaks of the XRD spectra may be attributed to the reduction in crystallite size. The average crystallite size can be estimated using Scherrer’s Equation [32]:

(5) $$D_{hkl}=\frac{k_{shape}\lambda }{\beta_{hkl}\cos\theta_{hkl}},$$

where *D_hkl_* is the crystallite size in the direction perpendicular to the (*hkl*) planes, *k_shape_* is the crystallite-shape factor which was taken to be 0.94 [4,33], *λ* is the wavelength of the X-ray, *β_hkl_* is the full-width at half-maximum of the X-ray diffraction peak in radians, and *θ_hkl_* is Bragg’s angle corresponding the (*hkl*) planes. The estimated average size of the CuIn_1−*x*_Ga*_x_*Se_2_ nanocrystals is listed in Table 1. Its value was in the range of 11.5–17.4 nm, which matched well with the TEM results (Figure 3).

### 3.2. Morphological Properties

TEM measurements were used to determine the morphology and size of the synthesized CuIn_1−*x*_Ga*_x_*Se_2_ nanocrystals. Figure 3a–l shows the TEM images of these samples. From Figure 3a–c, it can be seen that the CuIn_1−*x*_Ga*_x_*Se_2_ nanocrystals have irregular shapes as well as inhomogeneous sizes. The average size was around 15 nm for samples A and D, and around 12 nm for sample F. These values match those of the calculated results from the XRD data.

Higher resolution TEM images of the samples from A to I (Figure 3d–l) show well-defined lattice fringes in individual nanocrystals, which indicate good crystallinity. The average inter-plane spacings were in the range of 3.37–3.25 Å, and could be assigned to (112) lattice planes of chalcopyrite CuIn_1−*x*_Ga*_x_*Se_2_. There was a gradual decrement in these spacings with increasing Ga content. This is in agreement with the XRD results.

### 3.3. Determination of Band Gap Energy

**Figure 4:**
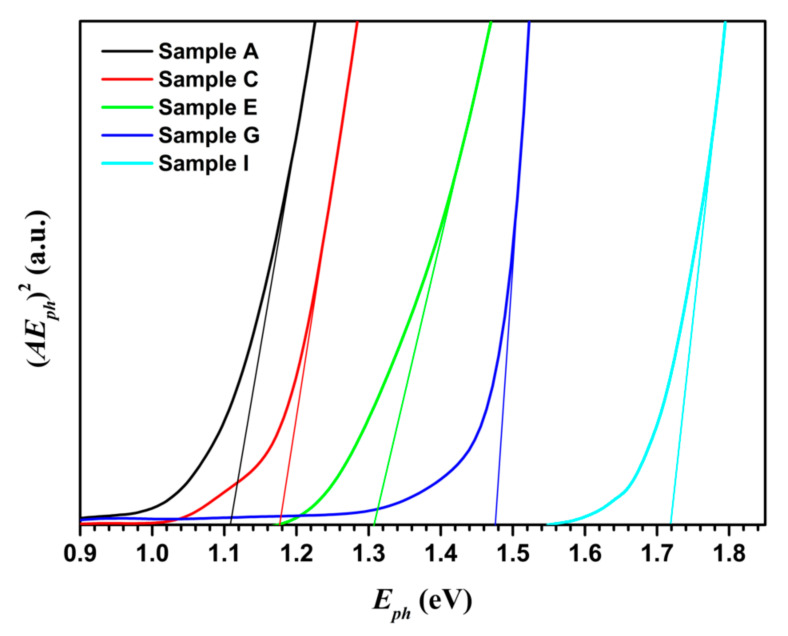
Tauc plot of (*AE_ph_*)^2^ versus *E_ph_* of CuIn_1−*x*_Ga*_x_*Se_2_ nanocrystals.

The absorption spectra of the CuIn_1−*x*_Ga*_x_*Se_2_ nanocrystals were measured at room temperature to investigate their absorption property and determine their optical band gap energy. For a direct-band gap semiconductor, the optical absorption near the band edge has the following behavior [34,35]:

(6) $$\alpha (E_{ph})=\left\{\begin{array}{cc} 0 & \mathrm{for}\;E_{ph}<E_{g},\\ B\frac{{\left(E_{ph}-E_{g}\right)}^{1/2}}{E_{ph}^{}} & \mathrm{for}\;E_{ph}\geq E_{g}, \end{array}\right.$$

where *α* is the absorption coefficient, *E_ph_* is the incident photon energy, *B* is a proportional constant, and *E_g_* is the band gap energy, respectively. Since the absorbance *A* of a sample is proportional to the absorption coefficient *α*, Equation (6) can be used to determine the optical band gap energy by an absorbance spectrum. Using the Tauc plot [36], as shown in Figure 4 for samples A, C, E, G, and I, the value of *E_g_* could be obtained through extrapolating the linear part of the (*AE_ph_*)^2^ vs. *E_ph_* curve at (*AE_ph_*)^2^ = 0. The band gap energy of the synthesized nanocrystals for samples A to I was found to be 1.108, 1.175, 1.177, 1.250, 1.307, 1.409, 1.476, 1.709, and 1.719 eV, respectively. The uncertainty of these values was about 0.01 eV. These values agree with those reported in other papers [4,21].

According to the effective mass approximation, there is a simple approximation formula which can be used to estimate the band gap energy of a nanocrystal of radius *R* [37,38]:

(7) $$E_{g}=E_{bulk}+\frac{{ћ}^{2}\pi^{2}}{2\mu R^{2}}-\frac{1.786e^{2}}{4\pi \varepsilon_{0}\varepsilon_{r}R}-0.248E_{Ry}^{*},$$

where *E_bulk_* is the band gap energy of bulk material, *μ* ≡ 1/(*m_e_*^−1^ + *m_h_^−^*^1^) is the reduced mass of an electron-hole pair (*m_e_* and *m_h_* are the effective masses of the electron and the hole, respectively), *ε_r_* is the dielectric constant, and E*_Ry_ is the effective Rydberg energy:

(8) $$E_{Ry}^{*}=\frac{\mu }{2{ћ}^{2}}{\left(\frac{e^{2}}{4\pi \varepsilon_{0}\varepsilon_{r}}\right)}^{2}.$$

The first term in Equation (7) represents the quantum confinement energy of an electron-hole pair, the second represents the Coulomb energy, and the third corresponds to the correlation between electron and hole.

The values found in the literature for the parameters in Equation (7) were $E_{bulk,{\mathrm{CuInSe}}_{2}}$= 1.07 eV [39], $m_{e,{\mathrm{CuInSe}}_{2}}$= 0.09*m*_0_ (where *m*_0_ is the electron rest mass), $m_{h,{\mathrm{CuInSe}}_{2}}$= 0.73*m*_0_ [40], $\varepsilon_{r,{\mathrm{CuInSe}}_{2}}$= 10.8 [41] for CuInSe_2_ and $E_{bulk,{\mathrm{CuGaSe}}_{2}}$= 1.68 eV [39], $m_{e,{\mathrm{CuGaSe}}_{2}}$= 0.14*m*_0_, $m_{h,{\mathrm{CuGaSe}}_{2}}$= 1.20*m*_0_ [40], $\varepsilon_{r,{\mathrm{CuGaSe}}_{2}}$= 10.6 [41] for CuGaSe_2_. For CuIn_1−*x*_Ga*_x_*Se_2_, $m_{e,{\mathrm{CuIn}}_{1-x}{\mathrm{Ga}}_{x}{\mathrm{Se}}_{2}}$,$m_{h,{\mathrm{CuIn}}_{1-x}{\mathrm{Ga}}_{x}{\mathrm{Se}}_{2}}$ and $\varepsilon_{r,{\mathrm{CuIn}}_{1-x}{\mathrm{Ga}}_{x}{\mathrm{Se}}_{2}}$ were assumed to be linearly dependent on *x*:

(9) $$\left\{\begin{array}{l} m_{e,{\mathrm{CuIn}}_{1-x}{\mathrm{Ga}}_{x}{\mathrm{Se}}_{2}}=(1-x)m_{e,{\mathrm{CuInSe}}_{2}}+xm_{e,{\mathrm{CuGaSe}}_{2}}\\ m_{h,{\mathrm{CuIn}}_{1-x}{\mathrm{Ga}}_{x}{\mathrm{Se}}_{2}}=(1-x)m_{h,{\mathrm{CuInSe}}_{2}}+xm_{h,{\mathrm{CuGaSe}}_{2}}\\ \varepsilon_{r,{\mathrm{CuIn}}_{1-x}{\mathrm{Ga}}_{x}{\mathrm{Se}}_{2}}=(1-x)\varepsilon_{r,{\mathrm{CuInSe}}_{2}}+x\varepsilon_{r,{\mathrm{CuGaSe}}_{2}} \end{array}\right.$$

and $E_{bulk,{\mathrm{CuIn}}_{1-x}{\mathrm{Ga}}_{x}{\mathrm{Se}}_{2}}$ could be calculated via the following equation [42,43]:

(10) $$E_{bulk,{\mathrm{CuIn}}_{1-x}{\mathrm{Ga}}_{x}{\mathrm{Se}}_{2}}=(1-x)E_{bulk,{\mathrm{CuInSe}}_{2}}+xE_{bulk,{\mathrm{CuGaSe}}_{2}}-0.19x(1-x)\;\mathrm{eV}.$$

**Figure 5:**
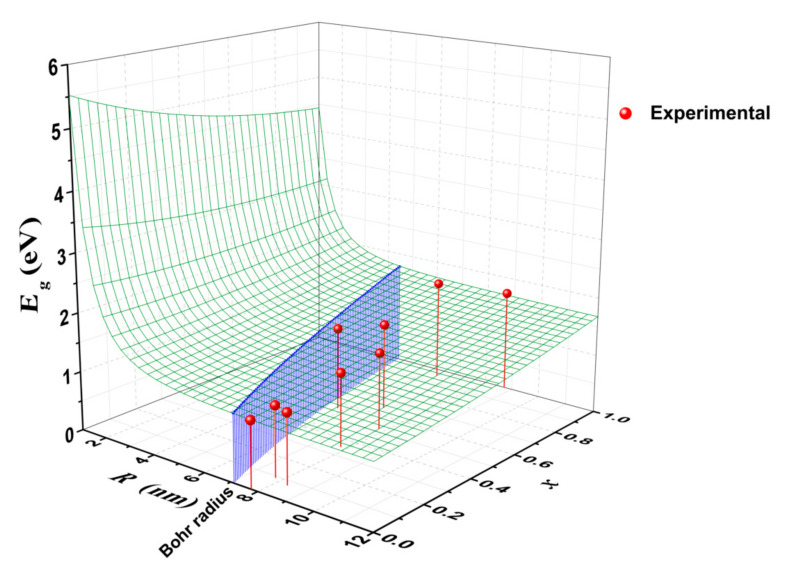
Band gap energy of CuIn_1−*x*_Ga*_x_*Se_2_ nanocrystals as a function of *x* and *R*.

Using Equations (7) to (10), the theoretical band gap energy of a CuIn_1−*x*_Ga*_x_*Se_2_ nanocrystal was calculated and displayed in Figure 5 as a function of *x* and *R*. For reference, the value of exciton Bohr radius of the crystal:

(11) $$a_{B}=\frac{4\pi \varepsilon_{0}\varepsilon_{r}}{\mu e^{2}}$$

is also shown in Figure 5 as a function of *x*. When the crystal radius was much smaller than the Bohr radius, the band gap energy decreased rapidly as *R* increased, and decreased gradually as *x* increased. However, when the crystal radius was larger than the Bohr radius, the band gap energy decreased slowly as *R* increased, and increased gradually as *x* increased.

The experimental values of band gap energy obtained from the absorption measurements are also shown in Figure 5 for comparison. They match well with the theoretically expected values. Since the radii of the synthesized nanocrystals were larger than their corresponding exciton Bohr radii, which were in the range of 4.47–7.13 nm, it would be expected that the primary factor that governs the band gap energy would be their composition ratio of In to Ga, with their size being a secondary factor. Therefore, the experimental band gap energy increased as *x* increased.

### 3.4. Raman Analysis

**Figure 6:**
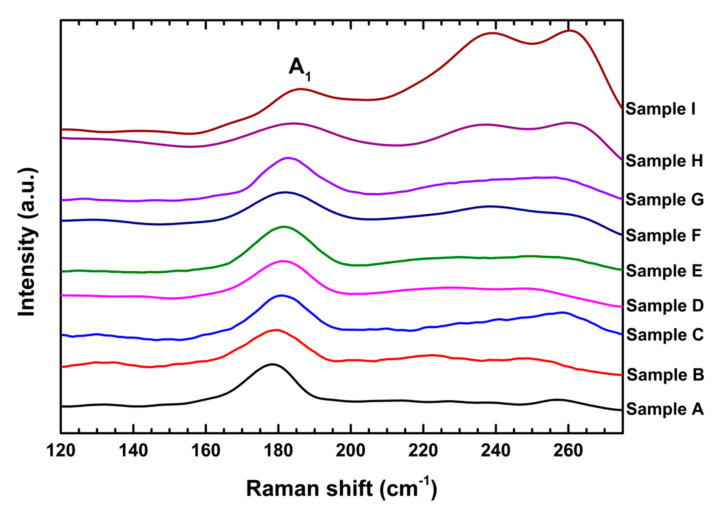
Raman spectra of CuIn_1−*x*_Ga*_x_*Se_2_ nanocrystals.

The Raman spectra of the CuIn_1−*x*_Ga_*x*_Se_2_ nanocrystals recorded at room temperature are shown in Figure 6. The intense peaks observed at around 180 cm^−1^ were due to the A_1_ mode [44], which is the strongest signal generally observed in the Raman spectra of I–III–VI_2_ chalcopyrite compounds. This mode resulted from the vibrations of the two pairs of Se anions in a unit cell of a chalcopyrite structure, i.e., one in the direction of the a-axis and the other in the direction of the b-axis [45]. The peaks observed at around 233 cm^−1^ may have been due to the B_2_/E modes of the chalcopyrite crystals [44,45], which represented the vibrations of anions and cations together. There were additional broad peaks at around 260 cm^−1^ which could be assigned to the A_1_ mode of Cu-Se compounds [46]. These indicated the appearance of Cu_x_Se in our samples.

**Figure 7:**
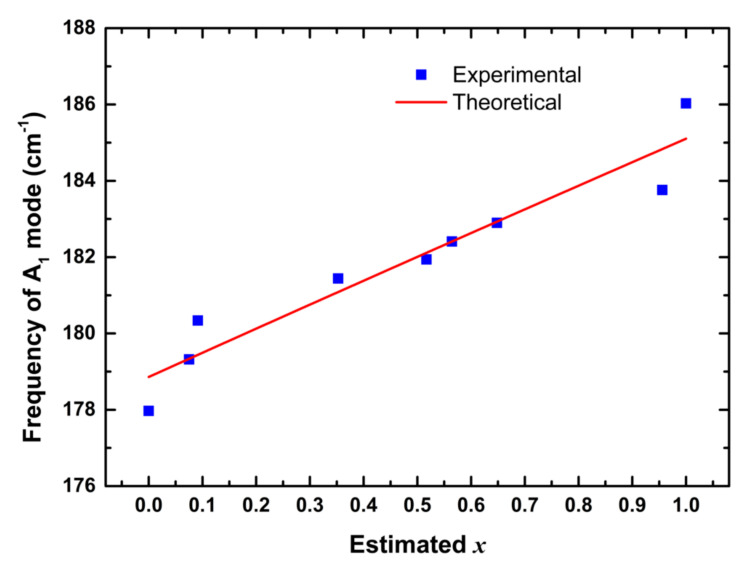
Frequency of A_1_ mode of CuIn_1−*x*_Ga*_x_*Se_2_ nanocrystals as a function of estimated *x*.

Figure 7 shows the frequency of A_1_ mode as a function of the estimated *x*. One can see that the A_1_ mode frequency shifted from 178 to 186 cm^−1^ with increasing Ga content. This is consistent with previous reports [43,46,47,48]. The A_1_ mode represented the vibration of the Se anion in the *xy* plane with the cations at rest. Accordingly, its frequency could be expressed by:

(12) $$\omega_{A_{1}}=\sqrt{\frac{k_{\mathrm{Cu}-\mathrm{Se}}+(1-x)k_{\mathrm{In}-\mathrm{Se}}+xk_{\mathrm{Ga}-\mathrm{Se}}}{M_{\mathrm{Se}}}}$$

where *M*_Se_ is the mass of Se anion, and *k*_Cu-Se,_ *k*_In-Se,_ and *k*_Ga-Se_ are the force constants of the bonds between the Se anions and the Cu, In, and Ga cations, respectively.

Assuming that the force constants were simply proportional to the inverse of the distances between the cations and anions, Equation (12) could be rewritten as:

(13) $$\omega_{A_{1}}=\sqrt{C_{\mathrm{Cu}-\mathrm{Se}}\frac{1}{d_{\mathrm{Cu}-\mathrm{Se}}}+C_{\mathrm{In}-\mathrm{Se}}\frac{1-x}{d_{\mathrm{In}-\mathrm{Se}}}+C_{\mathrm{Ga}-\mathrm{Se}}\frac{x}{d_{\mathrm{Ga}-\mathrm{Se}}}}$$

where *C*_Cu-Se_, *C*_In-Se_, and *C*_Ga-Se_ are constants, and *d*_Cu-Se_, *d*_In-Se_, and *d*_Ga-Se_ are the interionic distances between the Se anions and the Cu, In, and Ga cations, respectively. Using the ionic radii of Cu (0.96 Å), In (0.81 Å), Ga (0.62 Å), Se (1.98 Å) [27], and the correction Δ_N_ = −0.11 Å for coordination number N = 4 [49], we have *d*_Cu-Se_ = 2.83 Å, *d*_In-Se_ = 2.68 Å, *d*_Ga-Se_ = 2.49 Å.

The theoretical plot of the frequency of A_1_ mode versus *x*, obtained from Equation (13), shows the same trend as the experimental data. Ga ions are smaller than In ions, which resulted in *d*_Ga-Se_ < *d*_In-Se_ and *k*_Ga-Se_ > *k*_In-Se_. Accordingly, the frequency of A_1_ mode increased with increasing Ga content.

## 4. Conclusions

In conclusion, CuIn_1−*x*_Ga_*x*_Se_2_ nanocrystals with varying *x* value from 0 to 1 were synthesized by a simple solution-based method. According to XRD and TEM results, their particle size was in the range of 11.5–17.4 nm. The nanocrystals had a chalcopyrite structure with decreased lattice constants as the Ga content increased. Using Vegard’s law, the *x* value of the nanocrystals was determined. From the absorption spectra, the band gap energy of these nanocrystals was determined, which was in the range of 1.11–1.72 eV as the Ga content increased. The variation of band gap energy was primarily governed by the Ga content rather than the crystal size, because the particle radii of these nanocrystals were larger than their corresponding exciton Bohr radii. Raman spectra indicated that the A1 optical vibrational mode of the crystals gradually shifted to a higher wavenumber as the Ga content increased. Assuming that the force constants of bonds were simply proportional to the inverse of distances between the cations and anions, a simple theoretical equation for the A_1_ mode frequency was proposed. The plot of this equation showed the same trend as the Raman data. Ga ions are smaller than In ions. Accordingly, the frequency of A_1_ mode increased with increasing Ga content.

## Figures and Tables

**Table 1:** Structural and optical parameters of CuIn_1−*x*_Ga*_x_*Se_2_ nanocrystals.

Sample	Nominal *x*	*d*_112_ (Å)	*a* (Å)	*c* (Å)	Estimated *x*	Crystallite Size (nm)	Gap Energy (eV)	Raman Shift (cm^−1^)
A	0	3.362	5.794	11.764	0	15.528	1.108 ± 0.01	177.97 ± 1.52
B	0.125	3.353	5.782	11.721	0.075	16.711	1.175 ± 0.01	179.32 ± 0.53
C	0.25	3.351	5.779	11.712	0.091	15.533	1.177 ± 0.01	180.34 ± 1.29
D	0.375	3.323	5.745	11.555	0.353	15.530	1.250 ± 0.01	181.44 ± 1.10
E	0.5	3.311	5.743	11.438	0.517	15.537	1.307 ± 0.01	181.94 ± 0.48
F	0.625	3.302	5.722	11.424	0.564	11.501	1.409 ± 0.01	182.41 ± 0.53
G	0.75	3.288	5.693	11.391	0.648	13.659	1.476 ± 0.01	182.90 ± 0.48
H	0.875	3.247	5.628	11.232	0.956	12.818	1.709 ± 0.01	183.76 ± 0.68
I	1	3.237	5.604	11.223	1	17.,442	1.719 ± 0.01	186.03 ± 0.86
